# Dietary Lipid Quantity and Quality Modulate the Postprandial Metabolomic Profile in Patients with Metabolic Syndrome

**DOI:** 10.3390/nu16244267

**Published:** 2024-12-11

**Authors:** Marina Mora-Ortiz, Elena M. Yubero-Serrano, Feliciano Priego-Capote, Francisco M. Gutierrez-Mariscal, Juan F. Alcala-Diaz, José D. Torres-Peña, Antonio P. Arenas de-Larriva, Javier Delgado-Lista, Pablo Perez-Martinez, Helen M. Roche, José López-Miranda

**Affiliations:** 1 Lipids and Atherosclerosis Unit, Internal Medicine Unit, Reina Sofia University Hospital, 14004 Cordoba, Spain; elena.yubero@imibic.org (E.M.Y.-S.); francisco.gutierrez@imibic.org (F.M.G.-M.); juanf.alcala.sspa@juntadeandalucia.es (J.F.A.-D.); h52arlaa@uco.es (A.P.A.d.-L.); md1delij@uco.es (J.D.-L.); pablopermar@yahoo.es (P.P.-M.); 2 Maimonides Biomedical Research Institute of Cordoba (IMIBIC), 14004 Cordoba, Spain; 3 Department of Medical and Surgical Sciences, Universidad de Córdoba, 14071 Cordoba, Spain; 4 CIBER Fisiopatología de la Obesidad y Nutrición (CIBEROBN), Instituto de Salud Carlos III, 28029 Madrid, Spain; 5 Department of Food and Health, Instituto de la Grasa, Spanish National Research Council (CSIC), 28006 Seville, Spain; 6 Departamento de Química Analítica y Nanoquímica, Edificio Marie Curie (anexo), Universidad de Córdoba, 14071 Cordoba, Spain; q72prcaf@uco.es; 7 CIBER de Fragilidad y Envejecimiento Saludable (CIBERFES), Instituto de Salud Carlos III, 28029 Madrid, Spain; 8 Nutrigenomics Research Group, University College Dublin Conway Institute, School of Public Health, University College Dublin, D04 V1W8 Dublin, Ireland; helen.roche@ucd.ie; 9 Institute for Global Food Security, Queen’s University Belfast, Belfast BT7 1NN, UK

**Keywords:** metabolic syndrome, metabolomics, SFA, MUFAs, oxidative stress, inflammation

## Abstract

The literature on the postprandial metabolic changes in individuals with Metabolic Syndrome (MetS) remains limited, despite the fact that postprandial states represent the most common physiological condition in Western societies. Background/Objectives: The objective of this study was to investigate the plasma metabolomics profile in both fasting and postprandial states following a high-fat challenge in individuals with MetS who consumed diets with varying quantities and qualities of dietary fat over 12 weeks. Methods: Seventy-five patients with MetS (28 males and 47 females) from the Spanish LIPGENE cohort were included in the study. MetS patients were randomly stratified to follow one of four dietary interventions (isoenergetic diets) for a 12-week long-term study. The four diets were high in saturated fatty acids and high in monounsaturated fatty acids (HSFA and HMUFA), low-fat high-complex carbohydrates (LFHCC), and LFHCC supplemented with *n*-3. The metabolomics analysis of plasma samples was carried out using Liquid Chromatography Time-of-Flight Mass Spectrometry (LC-TOF/MS). Results: We observed a decrease in inflammation biomarkers, including acetylcarnitine and L-carnitine during the fasting state and hexanoyl-L-carnitine and isobutyryl-L-carnitine during the postprandial period, mediated by the replacement of HSFA with HMUFA. Additionally, antioxidant compounds such as 4-hydroxybenzaldehyde and L-valine were expressed at higher levels after consumption of the HMUFA diet compared to the HSFA diet. HSFA also presented altered levels of phosphatidylcholine, a metabolite previously linked with insulin resistance. Conclusions: These findings suggest that replacing HSFA with HMUFA may reduce inflammation and improve antioxidant profiles, supporting the potential for tailored dietary interventions in individuals with MetS.

## 1. Introduction

Metabolic syndrome (MetS), a metabolic disorder predominantly affecting the postprandial state, is considered the anteroom of noncommunicable diseases such as cardiovascular diseases (CVD) and type 2 diabetes (T2D) [1,2,3,4]. Some authors have estimated that the incidence of MetS worldwide is rising and reaching epidemic proportions with a prevalence of 25% [5], which is mainly due to complex interactions between genetic and environmental factors, including diet and, particularly, the quality of dietary fats [6]. Despite the epidemic impact of MetS on the health of the population, the pathogenicity and aetiology remain poorly understood. However, scientific evidence points out that postprandial metabolism has a determinant role in the progression of the disease, influencing atherosclerosis development, inflammation, insulin sensitivity, and other risk factors associated with MetS [7,8]. The postprandial state—which is physiologically altered in MetS—is the predominant state of people in Western societies [9]. However, most nutritional studies have focused on the hormonal and metabolic fasting state, and little is known about the metabolomic changes occurring in the postprandial state [1,9]. Therefore, there is a pressing need to understand the metabolic changes occurring during the postprandial state to establish preventive and effective strategies that may reduce MetS incidence and, thus, the development of associated diseases.

A broad range of metabolites—biogenic amines, amino acids, branched-chain amino acids, and aromatic amines—have been previously studied as potential biomarkers of the underlying pathways in the development of the MetS, characterizing it as a subclinical pro-inflammatory condition even in nascent stages [10]. In this sense, metabolomics could provide insight into metabolic adaptive alterations occurring in biological fluids, which are most closely related to metabolic endpoints or phenotypic characteristics. However, a small number of investigations have interrogated metabolomics modulations in MetS, particularly those associated with dietary modifications, limiting the understanding of the implications of dietary habits in the metabolomics modulations in MetS. Investigating the changes in metabolite levels related to the interaction between MetS and diet could help clarify how diet modifies metabolism in MetS patients and guide the development of new therapeutic dietary strategies.

The LIPGENE study is a dietary intervention trial aimed at analyzing the effects of reducing dietary saturated fatty acids (SFA) through isoenergetic modifications in both the quality and quantity of dietary fats in both fasting and postprandial states among patients with MetS [11,12]. Previous findings from the LIPGENE study showed that replacing SFA with polyunsaturated fatty acids (PUFAs) reduced several risk factors associated with MetS [13]. Moreover, the replacement of SFA with monounsaturated fatty acids (MUFA) also modulated different pathways underlying MetS development. Specifically, improvements were observed in postprandial endothelial cell function, along with reductions in pro-inflammatory markers and oxidative stress, owing to an enhanced postprandial antioxidant response in adipose tissue [13,14].

Research around MetS has been devoted to the implications of dietary interventions, but limited attention has been given to the underlying metabolomics profile, particularly during the postprandial state. In this study, we hypothesized that diet—specifically, the quantity and quality of dietary fats—plays a crucial role in shaping the metabolomic profile of MetS patients. Therefore, we aim to explore the plasma metabolomics profile during fasting and postprandial states, examining the effects of replacing high-saturated fatty acids (HSFA) with high-monounsaturated fatty acids (HMUFA) or low-fat, high-complex carbohydrate (LFHCC) diets, including an *n*-3 supplementation arm. This understanding could pave the way for personalized medicine by identifying the most effective dietary strategies tailored to improve the management of MetS.

## 2. Materials and Methods

### 2.1. Study Population

The present study was conducted within the framework of “The LIPGENE study (Diet, genomics and the metabolic syndrome: integrated nutrition, agro-food, social and economic analysis)”, a Framework 6 Integrated Project funded by the European Union [12]. Seventy-five patients with MetS (28 males and 47 females) from the Spanish LIPGENE cohort were included in the study. MetS was defined based on published criteria [15] that also aligned with the LIPGENE inclusion and exclusion criteria [7]. All participants gave written, informed consent and underwent a comprehensive medical history, physical examination, and clinical chemistry analysis before enrollment. This study was carried out in the Lipid and Atherosclerosis Research Unit at the Reina Sofia University Hospital between February 2005 and April 2006. The experimental protocol was approved by the local ethics committee according to the Helsinki Declaration. LIPGENE was registered with the US National Library of Medicine Clinical Trials registry (NCT00429195). The seventy-five patients with MetS used in this study are part of the Spanish LIPGENE cohort. This study was approved by the Comité Ético de Investigación Clínica del Hospital Universitario “Reina Sofía” (Ethics Committee) under the Project Identification Code FP6-2002-FOOD-1-505944 (date of approval: 10 February 2004). While the study population and initial data collection occurred between 2005 and 2006, the stored biological samples have since been used for ongoing research [16,17,18]. These follow-up studies, including the current work, leverage emerging analytical technologies to address new research questions, further maximizing the scientific output of the LIPGENE cohort.

### 2.2. Participants

The eligibility criteria for the study included volunteers with a body mass index (BMI) of 20–40 kg/m^2^ and aged 30–70 years following the National Cholesterol Education Program (NCEP) criteria for MetS [7], and the presence of MetS when participants had at least three of the following five inclusion considerations: (i) serum triglyceride levels ≥ 1.5 mmol/L, (ii) fasting plasma glucose levels 5.5–7.0 mmol/L, (iii) serum high-density lipoprotein cholesterol levels < 1.0 mmol/L in males and <1.3 mmol/L in females, (iv) elevated blood pressure (BP), defined as systolic BP ≥ 130 mmHg or diastolic BP ≥ 85 mmHg, or the use of BP-lowering medication, and (v) waist circumference > 102 cm in males and >88 cm in females. A more detailed description of this cohort of patients is available in previous publications [7,12,19].

### 2.3. Study Design: Randomization and Intervention

MetS patients were randomly assigned to follow one of four dietary interventions (isoenergetic diets) for a 12-week dietary intervention (Supplementary Material Figure S1). A full description of the dietary targets has been provided previously [8,20].

- High-SFA diet [(HSFA, 38% energy), where 16% energy came from the SFA, 12% MUFAs, and 6% PUFAs; *n* = 17].
- High-MUFA diet [(HMUFA, 38% energy), where MUFA provided 20% energy; 8% came from SFAs, and 6% from PUFAs; *n* = 18].
- LFHCC diet (low-fat and high in complex-carbohydrates diet) [28% energy: 8% SFAs, 11% MUFAs, and 6% PUFAs, plus a 1.24 g/d control high–oleic acid sunflower seed oil capsule (4 × 1 g capsules/d); *n* = 20].
- LFHCC *n*-3 diet [28% energy: 8% SFAs, 11% MUFAs, and 6% PUFAs, plus 1.24 g/d long-chain *n*-3 PUFAs [ratio of 1.4 eicosapentaenoic acid (EPA): 1 docosahexaenoic acid (DHA); *n* = 20].

The fat challenge, which involved a fat overload providing 65% of total energy from fat, was used to study postprandial responses after long-term adaptation to the corresponding diet. The intervention meals were administered post-intervention (at week 12) in the clinical center, with participants fasting for 12 h prior and refraining from smoking during this period and alcohol for the previous 7 days. Test meals provided a consistent amount of fat (0.7 g/kg body weight), energy (40.2 kJ/kg body weight), cholesterol (5 mg/kg body weight), fiber, and vitamin A (62.9 μmol vitamin A/m^2^ body surface area). The study utilized a range of dietary profiles: high-SFA (HSFA, 38% energy from saturated fat), based on butter, whole milk, white bread, and eggs; high-MUFA (HMUFA, 43% energy from monounsaturated fat), using olive oil, skimmed milk, white bread, eggs, egg yolks, and tomatoes; and low-fat high-carb (LFHCC) with placebo capsules and *n*-3 PUFA supplementation. Postprandial plasma samples were collected at baseline (fasting), 4 h, and 8 h after meal consumption and separated by centrifugation (1500× *g* for 15 min at 4 °C) for analysis within 1 h of extraction.

### 2.4. Dietary Adherence and Monitoring

Participants followed their assigned dietary protocols in a free-living environment, guided by a food-exchange model developed specifically for this study. This model allowed participants to replace habitual dietary fats (e.g., spreads, oils, snack items) with specially formulated study foods (e.g., spreads, oils, biscuits, and mayonnaise) that achieved the targeted fatty acid profiles while being palatable across diverse European populations. Participants were instructed to maintain other components of their diet (e.g., fruits, vegetables, proteins) as consistently as possible and were given detailed handbooks with food substitution tables, serving sizes, and sample recipes.

### 2.5. Compliance Assessment

Dietary adherence was closely monitored with biweekly food distributions (via collection or home delivery), 24 h dietary recalls, short food-use questionnaires, and 3-day weighed food diaries collected at baseline, week 6, and week 12. A points-based system was used to assess adherence, and body weight was also monitored to detect any unintended fluctuations. Compliance with the omega-3 fatty acid supplementation for the LFn-3 diet was checked through capsule counts. Adherence analysis showed significant changes in the dietary fatty acid profiles, aligning with the study’s intervention goals. These methods and outcomes have been documented in prior publications related to the LIPGENE project.

### 2.6. Dietary Assessment

Standard operating procedures and a common training protocol were employed to standardize dietary data and biological sample collection. Habitual dietary intake was assessed using a 3-day weighed food record (2 working days and 1 weekend day), which was the basis for the isoenergetic weight change (≤0.2 kg) used in the personalized fat modification [21]. Participants also completed a second 3-day food record at weeks 6 and 12 of the intervention period to ensure dietary adherence [12]. A dietary analysis program reflective of the food choices was used (Dietsource version 2.0). Volunteers were not advised to change their physical activity, smoking, or alcohol consumption habits; these were assessed using the health and lifestyle questionnaire at the beginning of the study and remained unaltered.

### 2.7. Sample Preparation and LC-TOF/MS Analysis

Of the 75 patients with MetS included in the study, 74 completed the follow-up dietary intervention study (both the long-term and postprandial period). Patients with at least 50% of the metabolites identified after LC-TOF/MS analysis were finally included in the statistical analysis (*n* = 69) (Supplementary Material Figure S1).

Plasma samples (100 µL), which had been previously immersed in a bath of ice, were treated with 300 µL of 1:2 methanol–chloroform. The mixture was shaken for 2 min and further stabilized for 3 min. The organic phase was separated after centrifugation for 5 min at 4 °C and 13,800× *g* in a thermostatic centrifuge, the Thermo Sorvall Legend Micro 21 R from Thermo (Thermo Fisher Scientific, Bremen, Germany). Both phases (aqueous and organic) were individually collected in vials and placed in the LC autosampler for subsequent analysis. All samples were analyzed in a 1200 Series LC system (Agilent Technologies, Waldbronn, Germany), which was coupled with an Agilent 6530 TOF mass spectrometer (Agilent Technologies, Santa Clara, CA, USA) equipped with a dual electrospray ionization source.

Chromatographic separation was performed using a Teknokroma Mediterranean Sea C18 analytical column (100 mm × 0.46 mm i.d., 3 μm particle size, from Barcelona, Spain) maintained at 25 °C. The mobile phases comprised (A) 0.1% formic acid in deionized water and (B) 0.1% formic acid in acetonitrile. The elution program was 0–2 min, 3% B; 2–30 min, 100% B. A post-run of 5 min was included to equilibrate the column. The flow rate was maintained at 0.8 mL/min. The volume of the injected sample was 10 μL, and the injector needle was washed 10 times with 70% methanol between injections. Therefore, the needle seat was flushed for 15 s at a flow rate of 4 mL/min with 70% methanol to avoid cross-contamination. Both the methanol and chloroform fractions were analyzed in both negative and positive ionization modes. The operating conditions in negative and positive ionization modes were: gas temperature, 325 °C; drying gas, nitrogen at 8 L/min; nebulizer pressure, 40 psi; sheath gas temperature, 350 °C; sheath gas flow, nitrogen at 11 L/min; capillary voltage, 4000 V; skimmer, 65 V; octopole radiofrequency voltage, 750 V; focusing voltage, 90 V. Data acquisition (2.5 spectra s–1; mass range 60–1100 *m*/*z*) was controlled using the Agilent Mass Hunter Workstation software, Version B.07.00. The instrument gave a typical resolution of 15,000 FWHM (Full Width at Half-Maximum) at *m*/*z* 112.9856 and 30,000 FWHM at *m*/*z* 1033.9881. To ensure the desired mass accuracy of recorded ions, continuous internal calibration was performed during analyses using signals at *m*/*z* 121.0509 (protonated purine) and *m*/*z* 922.0098 [protonated hexakis (1H, 1H, 3Htetrafluoropropoxy) phosphazine or HP-921] in positive ion mode. In negative ion mode, ions with m/z 119.0362 (proton-abstracted purine) and *m*/*z* 1033.988109 adducts of HP-921 were used. The instrument was calibrated and tuned according to procedures recommended by the manufacturer.

MassHunter Workstation software (version 3.01 Qualitative Analysis, Agilent Technologies, Santa Clara, CA, USA) was used for processing all data obtained by LC–TOF/MS in full scan MS mode. Treatment of raw data files began with the extraction of potential molecular features (MFs). Molecular features extraction (MFE) was based on an extraction algorithm that locates and groups all ions related to the same neutral molecule. This relationship is referred to as the covariance of peaks within the same chromatographic retention time, the charge-state envelope, isotopic distribution, and/or the presence of adducts and dimers. The MFE considered all ions exceeding 5000 counts, with a charge state limited to a maximum of two and a peak spacing tolerance of 0.0025 *m*/*z* (plus 50 ppm). Each feature was represented by a minimum of two ions. The extraction algorithm was based on a common organic model with chromatographic separation. The allowed positive ions were protonated species and sodium adducts (+H, +Na, +K), and the negative ions formed by formate adducts and proton losses (−H, −Cl, +HCOO). The neutral losses by dehydration and the loss of a phosphate or a methyl group were also included to identify features corresponding to the same molecule. Therefore, some ions with identical elution profiles and related *m*/*z* values (representing different adducts or isotopes of the same compound) were extracted as entities characterized by retention time (RT), intensity in the apex of chromatography peaks, and accurate mass. Background contributions were removed by subtracting MFs linked to plasticizers, solvent impurities, and other contaminants after analysis of the blank sample (methanol and chloroform) under identical instrument operation conditions. Therefore, data files were created in compound exchange format (.cef files) for each sample and exported into the Mass Profiler Professional (MPP) software package (version 2.0., Agilent Technologies, Santa Clara, CA, USA) for further processing. Finally, compound identification was performed using the METLIN Personal Metabolite Database and the Molecular Formula Generation algorithm (Agilent Technologies). The Human Metabolome Database (HMDB) was used to confirm and extend identification.

### 2.8. Data Processing and Statistical Analysis

Metabolites present in at least 80% of the samples were considered for further analysis, while individuals with less than 50% of the metabolites annotated were excluded. To facilitate predictive modeling, missing metabolite values were imputed by substituting them with half the average of the detected values for the respective metabolite. This imputation method is commonly used in metabolomics to address missing data while minimizing bias and ensuring robust statistical analyses.

The LC-MS (polar and apolar) matrix was imported into Matlab version R2015a (Mathworks, Natick, MA, USA) and analyzed using the statistical toolbox and algorithms from Korrigan Toolbox version 0.1 (Korrigan Sciences Ltd., Berkshire, UK). In Matlab, the matrix was normalized using a median-based probabilistic quotient method. The biostatistical pipeline for the multivariate statistical analysis of the samples considered a preliminary unsupervised Principal Component Analysis (PCA), followed by a supervised pairwise Orthogonal Projection to Latent Structures Discriminant Analysis (O-PLS DA) [22,23], which allowed the identification of specific modulations driven by the predictor included here, type of diet.

The goodness of prediction (Q^2^Y value) using 7-fold cross-validation was utilized to evaluate the resulting O-PLS DA models, and the overfit between R^2^Y and Q^2^Y was calculated. Only models with a positive Q^2^Y value and less than 50% overfit were further considered [24,25]. Metabolites from successful models were annotated and reported in the model loadings.

## 3. Results

The following section presents the key findings of our study, highlighting the metabolomic profiles observed and the impact of dietary interventions on patients with metabolic syndrome.

### 3.1. Postprandial Differences in MetS Patients over Time Compared to the Fasting Period

**Figure 1:**
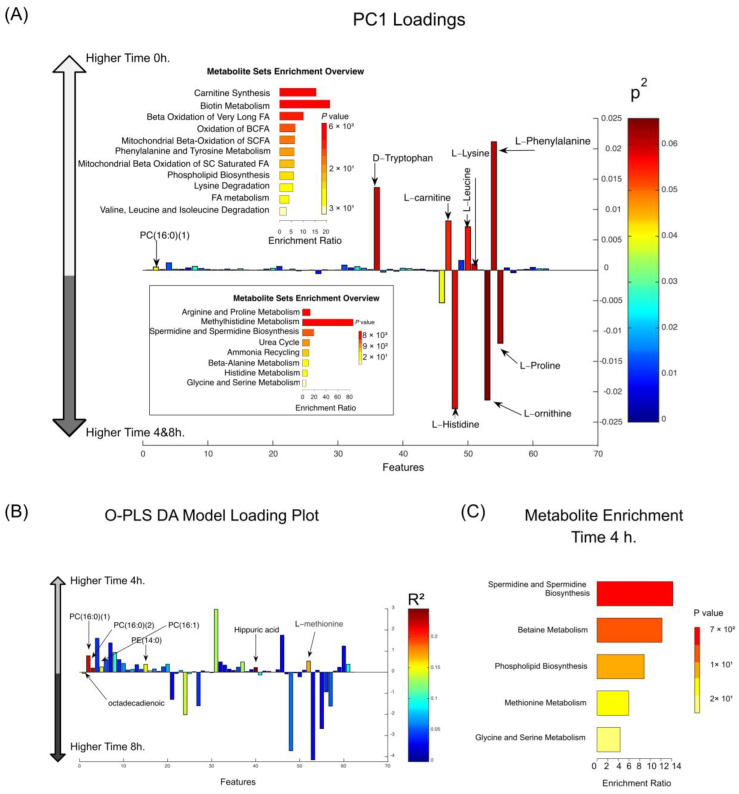
Postprandial metabolomic changes in MetS patients were analyzed across time and dietary interventions. (Panel (**A**)): Principal Component Analysis (PCA) loading plot for PC1. PC1 distinguished fasting (Time 0 h) from the postprandial period (Time 4 h and 8 h), accounting for 21% of the matrix variance. Metabolites with higher abundance at 0 h (fasting) are represented on the positive *Y*-axis, while those increased at 4 h and 8 h (postprandial states) are on the negative *Y*-axis. The accompanying “Metabolite Set Enrichment Overview” highlights pathways modulated during fasting (top) and postprandial states (bottom). (Panel (**B**)): O-PLS-DA loading plot, showing metabolites contributing to the separation between 4 h and 8 h. Metabolites enriched at 4 h are on the positive *Y*-axis, while those more abundant at 8 h are on the negative *Y*-axis. (Panel (**C**)): Metabolite enrichment analysis highlighting pathways significantly altered at 4 h relative to 8 h, focusing on specific metabolic processes such as lipid metabolism, phospholipid biosynthesis, and amino acid pathways. Note: Data were obtained using LC-TOF/MS metabolomic profiling. Four dietary interventions were analyzed at three time points (0 h, 4 h, 8 h), allowing for the identification of both fasting and postprandial metabolomic changes.

The unsupervised principal component analysis (PCA) of the postprandial period, within each dietary intervention, could distinguish between two major clusters along PC1, which accounted for 21% of variability. Time 0 h (fasting period) was plotted on the right-hand side, and times 4 h and 8 h (postprandial period) were plotted on the left-hand side (Supplementary Material Figure S2, Figure 1A). Carnitine synthesis and biotin metabolism were more expressed during fasting, with higher levels of L-carnitine, D-tryptophan, L-leucine, L-lysine, L-phenylalanine, and PC (16:0) higher expressed at time 0 h than later during the postprandial period. Conversely, arginine and proline metabolism and methyl-histidine metabolism were more stimulated at times 4 and 8 h, and the metabolites L-histidine, L-ornithine, and L-proline were found at higher levels at times 4 and 8 h (Figure 1A). The PCA did not show differences between time 4 and 8 h (postprandial period), and this sub-matrix of independent variables was further explored using an O-PLS DA model (R^2^Y = 0.40, Q^2^Y = 0.32; Figure 1B and Supplementary Material Figure S2). This O-PLS DA model showed that the spermidine biosynthesis metabolism was more stimulated at time 4 h, and the metabolites PC (16:0 and 16:1), PE (14:0), hippuric acid, and L-methionine were increased at time 4 h than later, at time 8 h, where only octadecadienoic acid was found at higher levels than in time 4 h (Figure 1B). Furthermore, the enrichment analysis showed significant alterations in the spermidine biosynthesis pathway followed by differences in betaine metabolism at time 4 h (Figure 1C).

**Figure 2:**
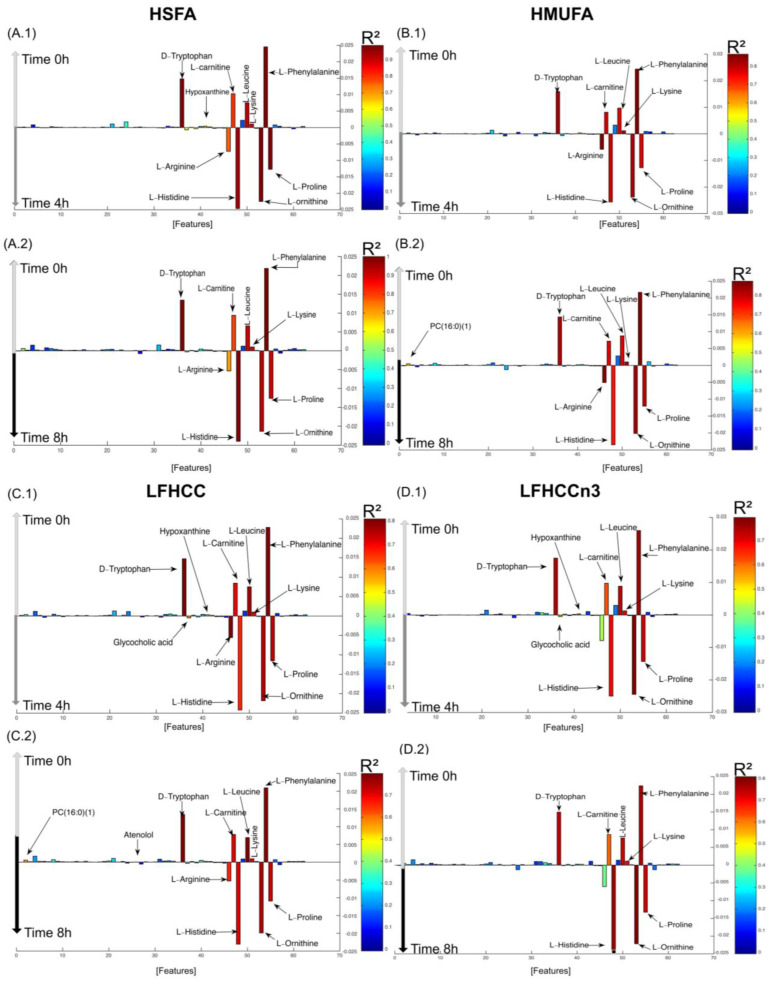
Postprandial changes in metabolic syndrome (MetS) patients at different time points within each dietary intervention. (Panels (**A1**,**A2**)) High Saturated Fatty Acid (HSFA) diet comparing baseline (0 h) against 4 h postprandial (Panels (**A1**)) and baseline against 8 h (Panel (**A2**)). (Panels (**B1**,**B2**)) High Mono-unsaturated Fatty Acid (HMUFA) diet comparing baseline (0 h) against 4 h postprandial (Panels (**B1**)) and baseline against 8 h (Panel (**B2**)). (Panels (**C1**,**C2**)): low-fat high-complex carbohydrate (LFHCC) diet comparing baseline (0 h) against 4 h postprandial (Panels (**C1**)), and baseline against 8 h (Panel (**C2**)): low-fat high-complex carbohydrate supplemented with omega-3 fatty acids (LFHCCn3) diet comparing baseline (0 h) against 4 h postprandial (Panels (**D1**)), and baseline against 8 h (Panel (**D2**)). Metabolites are highlighted in red or blue to indicate increased or decreased abundance over time, respectively. Note: Data were obtained using LC-TOF/MS metabolomic profiling.

Subsequently, postprandial metabolomics changes were studied in more detail within each diet (Figure 2 and Supplementary Material Figure S2). The fasting state showed higher levels of D-tryptophan, hypoxanthine, L-carnitine, L-leucine, L-phenylalanine, and L-lysine (Figure 2(A1–D1)). Conversely, the postprandial state was characterized by higher levels of glycocholic acid (GA), L-histidine, L-ornithine, and L-proline. Some minor differences were observed in this general pattern depending on the diet. For example, hypoxanthine was found to be present at higher levels at time 0 h compared to 4 h in all diets except in the HMUFA diet, where no significant fluctuations in this metabolite were observed between both time points (Figure 2(B1)). Another specific within-diet difference was the modulation of GA, which was found expressed at higher levels in the two low-fat, high-complex carbohydrate diets (with and without supplementation with long-chain *n*–3 PUFAs) at time 4 h compared to fasting and not in the high-fat diets (Figure 2(C1,D1)). Further details about the score plots are provided in Supplementary Material Figure S2, Figure 2.

O-PLS DA models were also performed to compare time 0 h against 8 h (Figure 2(A2–D2)). The profiles were generally very similar to what was observed comparing time 0 h against 4 h, with some exceptions. The higher levels of hypoxanthine identified during fasting (time 0 h) compared to the postprandial period (time 4 h) were not observed here. Furthermore, PC (16:0) was found at higher levels in both the HMUFA and LFHCC diets. Atenolol was also found to be expressed at higher levels in the LFHCC diet when the fasting state is compared to time 8 h. Time 8 h showed higher levels in all the metabolites previously mentioned for time 4 h, except for the GA and isobutyl-L-carnitine.

### 3.2. Replacement of the Quality and Quantity of Dietary Fat-Induced Differences in the Metabolomic Profile During Fasting and the Postprandial Period in MetS

**Figure 3:**
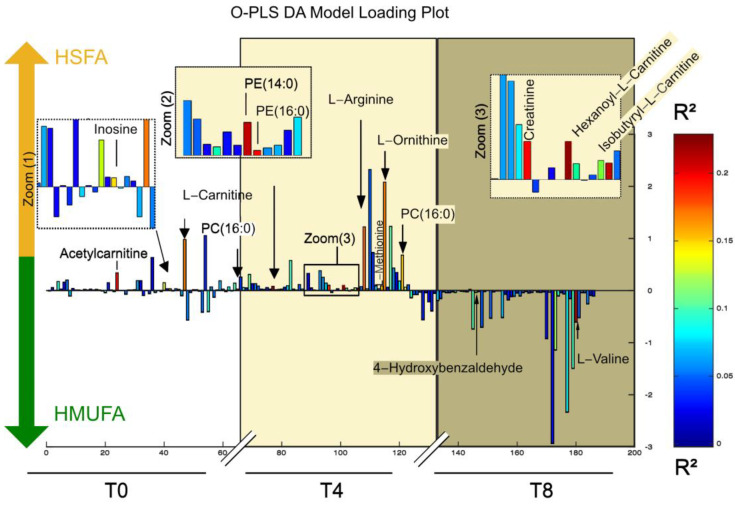
Postprandial metabolic differences between HSFA and HMUFA diets based on serum metabolite profiles. Loading plot from the OPLS-DA model (R^2^Y = 0.37, Q^2^Y = 0.04) showing postprandial metabolic changes at three time points: fasting (T0, white background), 4 h postprandial (T4, yellow background), and 8 h postprandial (T8, brown background). Metabolites contributing to the separation between diets and time points are highlighted. Note: Data were obtained using LC-TOF/MS metabolomic profiling.

**Figure 4:**
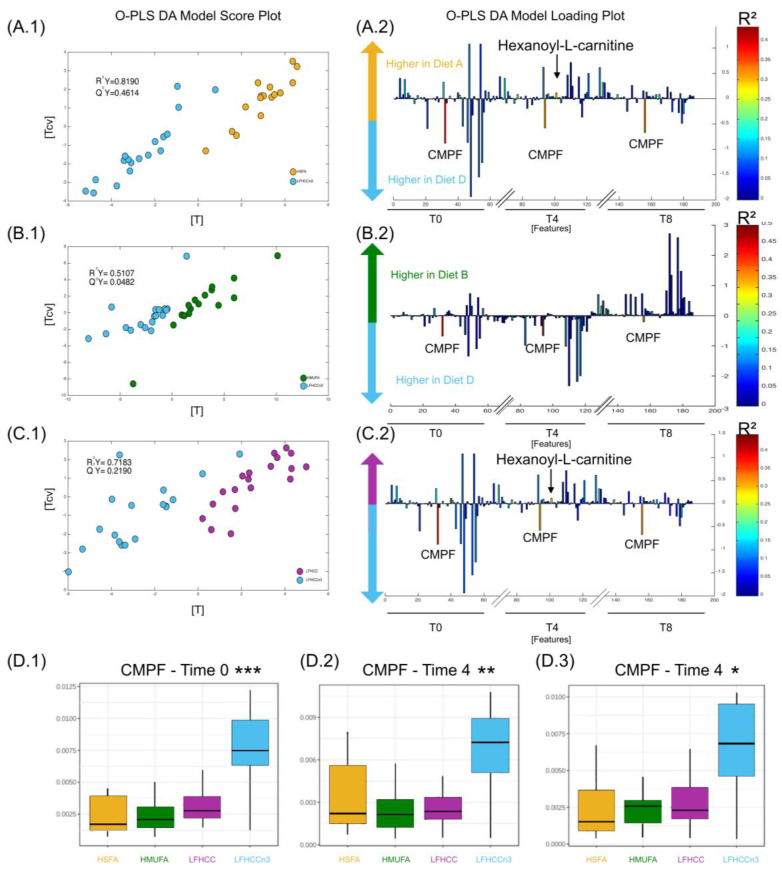
Metabolomics analysis of plasma samples comparing the LFHCCn-3 diet to HSFA, HMUFA, and LFHCC diets. (Panel (**A1**)): OPLS-DA score plot for the pairwise comparison of the High Saturated Fatty Acid (HSFA) diet (ochre) and the low-fat high-complex carbohydrate diet supplemented with omega-3 (LFHCCn-3, blue). The model was calculated using all plasma metabolites as independent variables and diet as the predictor (R^2^Y = 0.82, Q^2^Y = 0.42). (Panel (**A2**)): Loading plot showing the metabolites of interest identified as contributors to the model. (Panel (**B1**)): OPLS-DA score plot for the pairwise comparison of the High Mono-unsaturated Fatty Acid (HMUFA) diet (green) and the LFHCCn-3 diet. The model was generated using all plasma metabolites as independent variables and diet as the predictor. (Panel (**B2**)): Loading plot showing the metabolites driving the separation between these diets. (Panel (**C1**)): OPLS-DA score plot and associated loading plot for the pairwise comparison of the low-fat high-complex carbohydrate (LFHCC) diet (purple) and the LFHCCn-3 diet, calculated as described above. (Panel (**C2**)): Loading plot showing the metabolites of interest identified as contributors to the model presented on (Panel (**C1**)). (**D1**–**D3**): Boxplot illustrating the significantly higher concentrations of CMPF (3-carboxy-4-methyl-5-propyl-2-furanpropanoic acid) observed at fasting (0 h) and during the postprandial period (4 h and 8 h) for each diet in the interventional study. *: *p*-value = 0.05, **: *p*-value = 0.01, ***: *p*-value = 0.001.

Significant changes observed between the dietary pairwise comparisons are shown in Figure 3 and Figure 4.

The metabolic differences between the HSFA and HMUFA diets produced an O-PLS DA (R^2^ = 0.37, Q^2^ = 0.04) model that identified 15 metabolites of interest (Figure 3 and Supplementary Material Figure S2). In general, the HSFA was associated with a pro-inflammatory and pro-oxidative metabolism, both during the fasting and postprandial periods.

- The HSFA diet, compared to the HMUFA diet, had the strongest impact during the fasting period (time 0 h), with higher levels of metabolites associated with inflammation such as acetylcarnitine and L-carnitine. It also showed higher levels of inosine and phosphatidylcholine (PC) (16:0).
- The earliest postprandial period (time 4 h) showed higher levels of phosphatidylethanolamine (PE) (14:0), PC (16:0), hexanoyl-L-carnitine, isobutyryl-L-carnitine, creatinine, L-arginine, L-methionine, and L-ornithine. The HMUFA diet exhibited higher levels of two recognized molecules with antioxidant properties, L-valine, and 4-hydroxybenzaldehyde, during the late postprandial period (time 8 h).

We next carried out a metabolite enrichment analysis, which showed that (i) the beta-oxidation of very-long-chain fatty acids, (ii) the spermidine biosynthesis, (iii) the glycine and serine metabolism, and (iv) the oxidation of branched-chain fatty acids were important pathways upregulated by the HSFA diet (Supplementary Material Figure S2, Figure 4). Conversely, the HMUFA diet moderately influenced propanoate metabolism as well as the branched-chain amino acids metabolism (Supplementary Material Figure S2, Figure 4).

The pairwise comparison of the LFHCC *n*-3 diet against the HSFA diet (R^2^Y = 0.82, Q^2^Y = 0.46, Figure 4(A1,A2)) also identified higher levels of the inflammatory molecule hexanoyl-L-carnitine during the postprandial period (time 4 h) of the HSFA diet. This modulation was also observed in the pairwise comparison between LFHCC *n*-3 and the LFHCC diet (R^2^Y = 0.72; Q^2^Y = 0.22; Figure 4(C1,C2)). Interestingly, the pairwise comparison between the LFHCC *n*-3 diet and the HMUFA diet (R^2^Y = 0.51; Q^2^Y = 0.05, Q^2^Y = 0.05) did not identify additional metabolites apart from the CMPF previously mentioned, which means that both diets (i.e., LFHCC *n*-3 and HMUFA) had comparable levels of hexanoyl-L-carnitine. Finally, the boxplots from (Figure 4(D1–D3)) show an average increase in CMPF in the LFHCCn-3 diet compared to the rest of the diets.

## 4. Discussion

There is an urgent need to understand the underlying pathophysiology of MetS, particularly regarding postprandial responsiveness to dietary interventions, which represent the dominant physiological state in Western society. In recent years, there has been increasing interest in how diet influences metabolic responses after food intake, with a growing body of evidence suggesting that postprandial metabolism plays a critical role in disease progression and management. In this study, we employed untargeted LC-MS metabolomics to discover fasting and postprandial metabolomics fluctuations in MetS patients, identifying biomarkers associated with dietary interventions with different qualities and quantities of dietary fat. Our study contributes to the expanding knowledge of how different dietary patterns influence the metabolomic landscape, especially in patients with MetS. We also analyzed how specific dietary interventions influenced the metabolomic profiles of these patients.

The main results referred to the metabolic differences between postprandial and fasting states, with strong OPLS DA models highlighting the metabolic differences between both. These differences mainly referred to compounds related to the carnitine synthesis metabolism, the beta-oxidation of very long FA, and the biotin metabolism. In particular, the identified metabolites offer important clues to understanding how MetS patients manage lipid metabolism in different nutritional states. Comparison between dietary interventions showed that the supplementation with n3 was also metabolically significant, as well as the replacement of HSFA by HMUFA diet. These findings align with current research indicating the potential metabolic benefits of *n*-3 supplementation and MUFA-based diets in improving lipid profiles and reducing inflammation in MetS patients [19].

The metabolites identified in our study were predominantly linked to postprandial inflammation, antioxidant compounds, and lipoprotein synthesis. For example, LC, a metabolite associated with inflammation, was identified at higher levels in the HSFA group. Recent evidence suggests that LC, a biogenic amine found in meat products, had a 2.5-fold median increase in individuals with an early stage of MetS and was inversely correlated with anti-inflammatory compounds such as adipokine and adiponectin [26]. It was also evidenced in a different human study that LC was increased in a cohort when the patients also presented MetS [27]. These findings highlight the role of LC as a potential biomarker for inflammation and the early stages of MetS, suggesting its relevance as a target for dietary modulation. Several antioxidant compounds were modulated by substituting the HSFA diet with the HMUFA diet, including methionine, L-arginine, and L-ornithine. Although limited, prior studies have reported elevated methionine levels in Zucker-obese rats with the leptin mutation [28]. This finding aligns with the model proposed by Adams and colleagues, which links insulin to the accumulation of intermediary metabolites such as methionine due to reduced branched-chain α-keto acid dehydrogenase activity [29,30]. Similarly, arginine and ornithine have been associated with obesity and insulin deficiency, as evidenced in studies comparing obese versus lean individuals and insulin-deficient mouse models [31,32]. Conversely, MetS patients on an HMUFA diet exhibited elevated levels of 4-hydroxybenzaldehyde and L-valine during the postprandial period (8 h), both of which are recognized for their antioxidant properties [33,34]. This observation further supports the potential metabolic advantages of a MUFA-enriched diet in reducing oxidative stress, which is a hallmark of MetS pathology. These findings collectively suggest that MetS following an HSFA diet exhibit a pronounced tendency toward inflammation and oxidation. Compared to those adhering to an HMUFA diet. This underscores the metabolic benefits of substituting SFA with MUFA [8].

This study also revealed a substantial metabolic impact from *n*-3 PUFA supplementation. The LFHCC-n3 differed markedly from all the other dietary interventions, primarily due to a statistically significant increase in the metabolite CMPF. Previous research showed that supplementation with Lovaza, an *n*-3 drug, enhances lipid metabolism, reduces hepatic steatosis, downregulates lipogenic gene expression, stimulates beta-oxidation, and improves insulin sensitivity in murine models [35]. Prentice and colleagues demonstrated that CMPF plays a mechanistic role in inhibiting acetyl-CoA-carboxylase activity, leading to a sustained downregulation of ACC1/2 expression and SREBP1c. These changes contribute to reduced hepatic lipid accumulation, modest increases in fasting blood glucose, decreased serum aspartate aminotransferase levels, and a reduction in fat mass over time. [7]. These findings further validate the role of CMPF in modulating lipid metabolism and suggest it as a potential therapeutic target for improving metabolic health in MetS patients. This finding is particularly relevant for MetS patients, as they often experience abnormal lipid storage in the liver and muscle due to dysregulated lipid metabolism [36,37]. In line with this, a prior publication from the LIPGENE study demonstrated that MetS patients consuming the LFHCC-*n*-3 diet exhibited significantly lower postprandial triglyceride concentrations compared to those following other dietary interventions [11]. Interestingly, previous findings from the LIPGENE study demonstrated that the LFHCC-*n*-3 diet reduced the risk of MetS [13]. In the current study, we observed that CMPF levels remained significantly elevated during the 8 h postprandial period, even with a modest intake of 1.24 g/d of *n*-3 fatty acids. This metabolite consistently yielded statistically significant models in pairwise comparisons against all other dietary interventions. These results suggest that even a relatively low dose of *n*-3 PUFAs could have a prolonged impact on postprandial metabolism, which warrants further investigation into the optimal dosage for therapeutic use in MetS. Further studies should explore the role of CMPF in managing MetS. Notably, CMPF is not classified as a toxic urinary metabolite and has been proposed as a potential biomarker for healthy dietary patterns. [38,39].

Interestingly, hypoxanthine levels were elevated during the 4 h postprandial period compared to the fasting state following all diets except the HMUFA diet. Hypoxanthine, a metabolite associated with purine degradation, has been linked in previous studies to training-induced adaptations in purine nucleotide metabolism, suggesting its potential relevance in metabolic and exercise physiology. [40]. This observation suggests a potential interaction between diet and purine metabolism in MetS patients, which could be explored further in future studies. Unfortunately, this study lacks metabolic data for urine samples, limiting the scope of interpretation for certain findings. Another notable observation was the postprandial increase of glycocholic acid (GA) following the two low-fat, high-complex carbohydrate diets (LFHCC and LFHCC *n*-3) at 4 h postprandially compared to the fasting state. GA, a bile acid conjugated with glycine and derived from cholic acid, is produced through the action of colonic microbiota enzymes, particularly from Bacteroides, Bifidobacterium, Clostridium, and Lactobacillus species [41]. This metabolite plays a critical role in fat emulsification, and its elevated postprandial levels may reflect the completion of the emulsification process for most dietary fats during this period. These findings highlight the interplay between dietary composition, gut microbiota activity, and lipid metabolism. The increase in GA following low-fat diets might suggest improved bile acid metabolism and fat emulsification, which could potentially enhance fat absorption and utilization. This warrants further investigation into the microbiome’s role in postprandial metabolism. Further studies should interrogate the microbiome of the patients and the link of this profile with the metabolic modulations observed during the postprandial period.

The LIPGENE project is a multicenter study with a well-characterized population that allows the extrapolation of the results to other European populations. However, a limitation of such trials is ensuring complete adherence to the dietary instructions given to the participants. Despite this common challenge, adherence to the recommended dietary patterns in the LIPGENE project was generally good, as assessed through routine dietary evaluations conducted during the intervention. One limitation of this study is the approach used for imputing missing metabolite values. We applied a commonly used method where missing values were replaced with half of the smallest annotated value for each metabolite [42]. This approach assumes that missingness is due to values falling below the instrument’s detection limit, which is a frequent cause in untargeted metabolomics datasets. While straightforward and widely implemented, which makes the results comparable to many studies in the field, this method can introduce biases, as it may overrepresent lower concentrations and does not account for missingness that could arise from other biological or technical factors. Another limitation of this study is that it was a secondary analysis of the LIPGENE project. As a result, more research specifically designed to explore metabolomic variations concerning dietary interventions will be necessary to confirm and expand upon these findings. Future work should focus on more comprehensive, targeted studies to validate the findings and further explore the metabolic changes induced by dietary interventions in MetS patients. Despite these limitations, this study offers important insights into the metabolic benefits of replacing saturated fatty acids with monounsaturated fatty acids, highlighting promising dietary strategies to reduce inflammation and oxidative stress in individuals with MetS and contributing to a growing body of evidence supporting personalized nutrition approaches for disease management.

## 5. Conclusions

In conclusion, replacing SFA with MUFA reduced the expression of biomarkers associated with inflammation and oxidative stress in MetS patients during both fasting and the postprandial period. Additionally, the HMUFA diet enhanced the levels of antioxidant metabolites and decreased phosphatidylcholine and phosphatidylethanolamine, two major components of plasma lipoproteins often linked with obesity, insulin resistance, and T2D when present in excessive amounts. Moreover, supplementation with *n*-3 in the LFCCH diet led to an increase in the postprandial concentration of CMPF, a metabolite known for its long-lasting protection against steatosis. More comprehensive studies focusing on dietary fat modifications are needed to further understand the role of fat quality and quantity in MetS risk factors and to inform nutrition-based therapeutic strategies for managing this disease.

## Data Availability

The data sets used or analyzed are available from the corresponding author upon request.

## Supplementary Materials

The following supporting information can be downloaded at https://www.mdpi.com/article/10.3390/nu16244267/s1, Figure S1: Multivariate Analysis of Fasting and Postprandial Metabolomic Profiles Across Dietary Interventions; Figure S2: Postprandial Metabolic Changes in MetS Patients at Different Time Points Across Dietary Interventions (Score Plots); Figure S3: Postprandial Metabolic Differences Between HSFA and HMUFA Diets Based on Serum Metabolite Profiles (Score Plot); Figure S4: Metabolite Enrichment Analysis of Postprandial Pathways Affected by HSFA and HMUFA Diets.
