# Supplementary material for: Dietary Lipid Quantity and Quality Modulate the Postprandial Metabolomic Profile in Patients with Metabolic Syndrome
Source: Nutrients. 2024 Dec 11;16(24):4267. doi: 10.3390/nu16244267 (PMC11677668; doi:10.3390/nu16244267)
Supplement: Supplementary file 1 [file nutrients-16-04267-s001.zip › nutrients-3345571-supplementary.pdf]

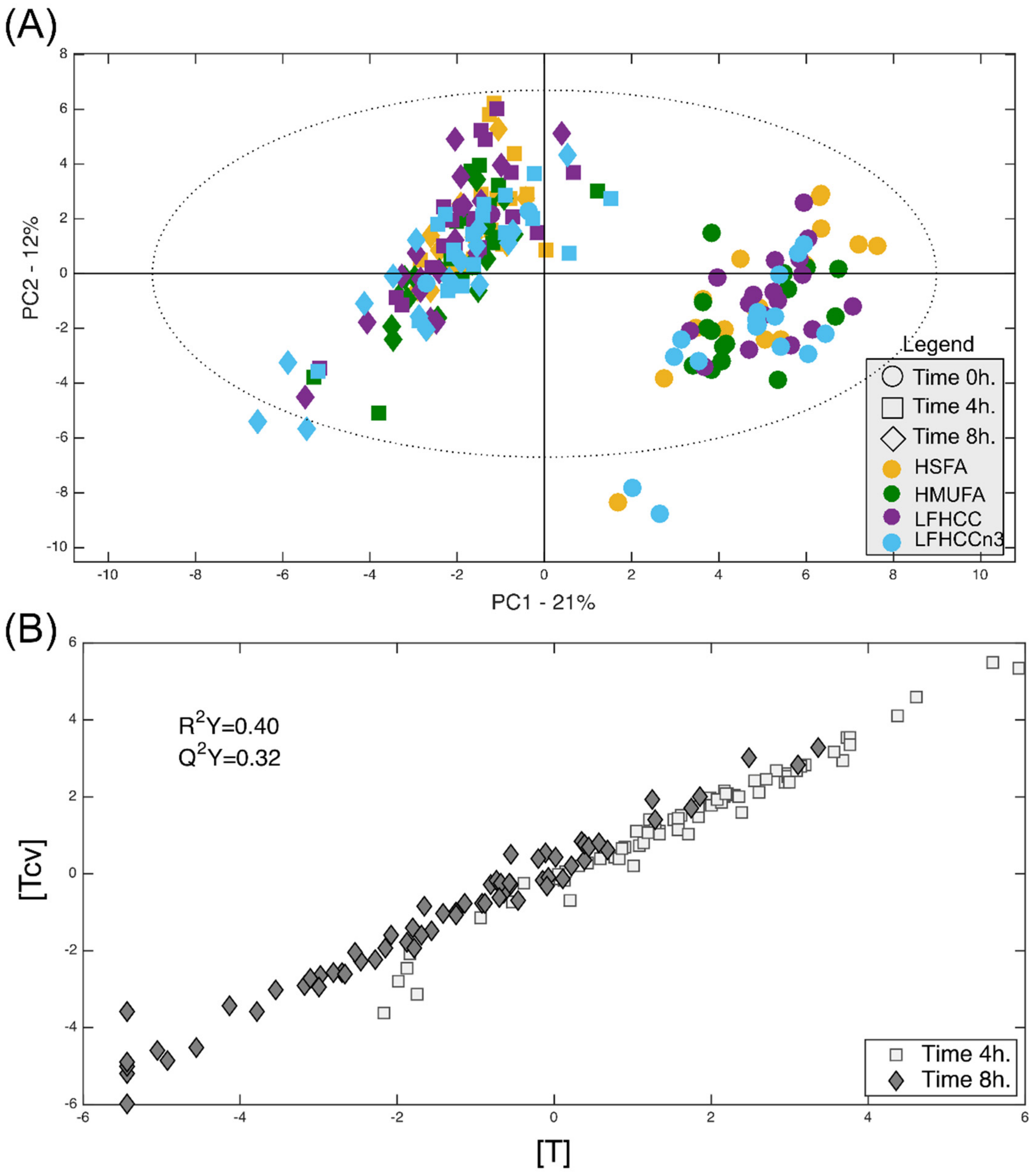

Supplementary Material- Figure S1: Multivariate Analysis of Fasting and Postprandial Metabolomic Profiles Across Dietary Interventions. (Panel A) Principal Component Analysis (PCA) of the postprandial period across dietary interventions identified two distinct clusters along PC1, which accounted for 21% of the variability. The fasting period (Time 0h) is depicted on the right-hand side, while the postprandial periods (Times 4h and 8h) are positioned on the left-hand side. **(Panel B)** Orthogonal Partial Least Squares Discriminant Analysis (O-PLS DA) model scores, with R^2Y=0.40 and Q^2Y=0.32, highlight further exploration of the sub-matrix of independent variables.

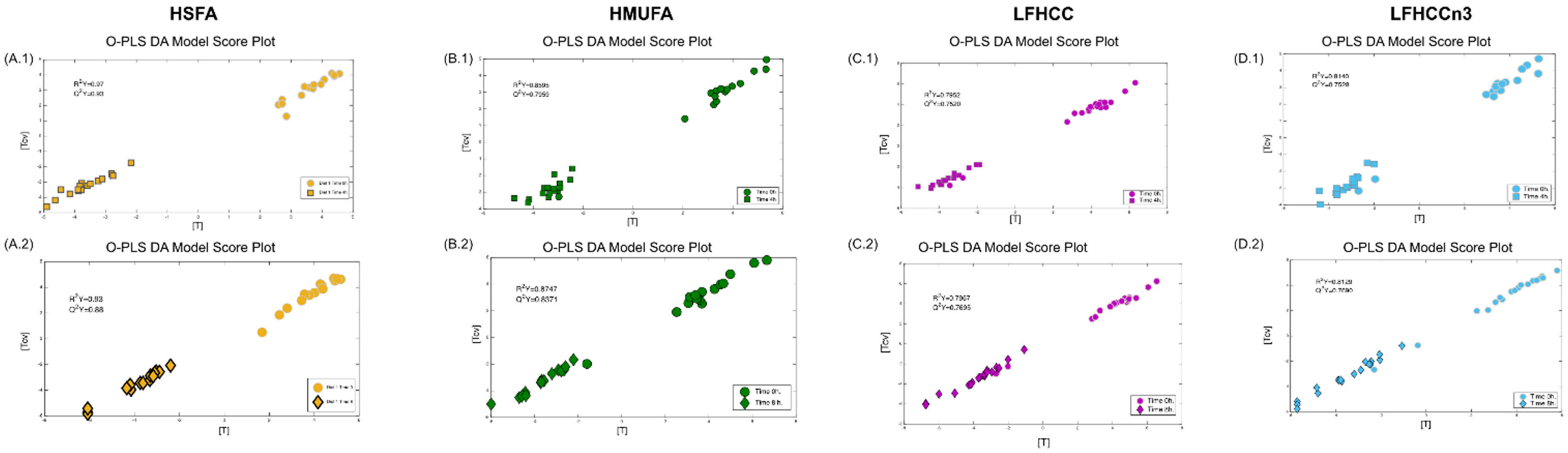

Figure S2. Postprandial Metabolic Changes in MetS Patients at Different Time Points Across Dietary Interventions (Score Plots). (Panels A.1 and A.2) Score plots for the High Saturated Fatty Acid (HSFA) diet comparing metabolomic profiles at baseline (0h) versus 4h postprandial (Panel A.1) and baseline versus 8h postprandial (Panel A.2).**(Panels B.1 and B.2)** Score plots for the High Mono-unsaturated Fatty Acid (HMUFA) diet comparing baseline (0h) versus 4h postprandial (Panel B.1) and baseline versus 8h postprandial (Panel B.2). **(Panels C.1 and C.2)** Score plots for the Low Fat High Complex Carbohydrate (LFHCC) diet comparing baseline (0h) versus 4h postprandial (Panel C.1) and baseline versus 8h postprandial (Panel C.2). **(Panels D.1 and D.2)** Score plots for the Low Fat High Complex Carbohydrate diet supplemented with omega-3 fatty acids (LFHCCn3), comparing baseline (0h) versus 4h postprandial (Panel D.1) and baseline versus 8h postprandial (Panel D.2). Data were generated through LC-TOF/MS metabolomic profiling.

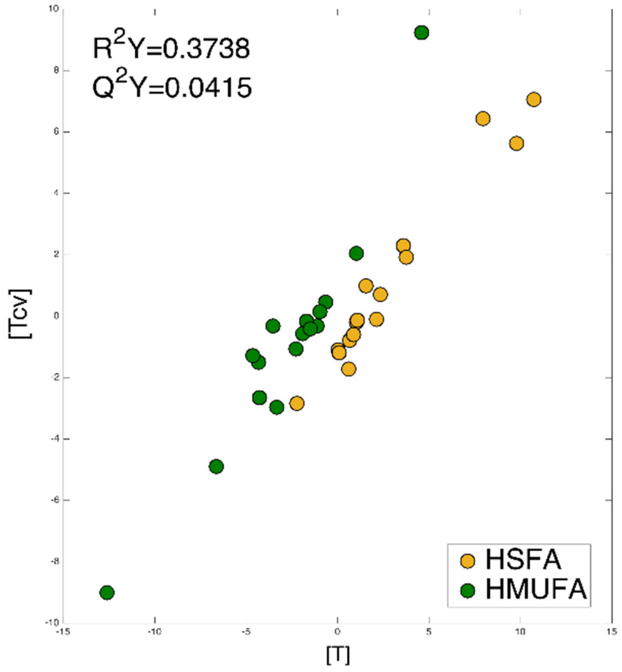

Figure S3. Postprandial Metabolic Differences Between HSFA and HMUFA Diets Based on Serum Metabolite Profiles (Score Plot). Score plot of the OPLS-DA model ($R^2Y = 0.37$, $Q^2Y = 0.04$) illustrating metabolic changes between the High Saturated Fatty Acid (HSFA) and High Mono-unsaturated Fatty Acid (HMUFA).

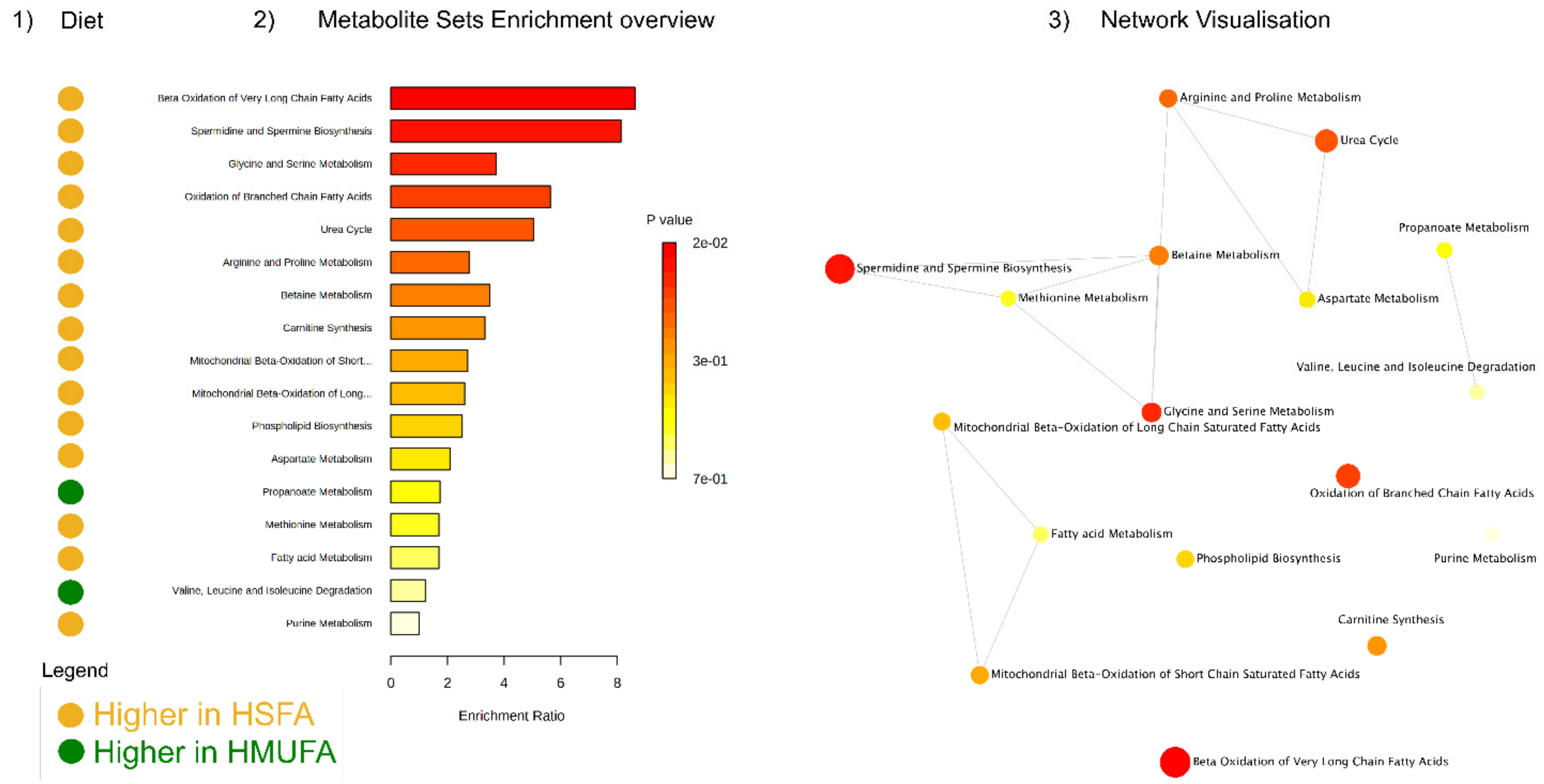

Figure S4. Metabolite Enrichment Analysis of Postprandial Pathways Affected by HSFA and HMUFA Diets. (**Panel 1**): Legend indicating metabolic pathways with increased activity associated with the High Saturated Fatty Acid (HSFA) diet (indicated in ochre) or the High Mono-unsaturated Fatty Acid (HMUFA) diet (indicated in green). **(Panel 2)**: Overview of metabolite set enrichment analysis highlighting the key pathways modulated by each dietary intervention. **(Panel 3)**: Network visualization chart illustrating the interconnections between enriched pathways and their comparative regulation under HSFA and HMUFA diets.
